# Molecular imaging of the human pulmonary vascular endothelium in pulmonary hypertension: a phase II safety and proof of principle trial

**DOI:** 10.1007/s00259-017-3655-y

**Published:** 2017-02-24

**Authors:** François Harel, David Langleben, Steve Provencher, Alain Fournier, Vincent Finnerty, Quang T. Nguyen, Myriam Letourneau, Xavier Levac, Gad Abikhzer, Jean Guimond, Asmaa Mansour, Marie-Claude Guertin, Jocelyn Dupuis

**Affiliations:** 1 Research Center, Montreal Heart Institute, 5000, Belanger Street, Montreal, QC H1T 1C8 Canada; 2 Department of Nuclear Medicine, Université de Montréal, Montréal, Québec Canada; 3 Lady Davis Institute and Jewish General Hospital, McGill University, Montréal, Québec Canada; 4 Institut Universitaire de Cardiologie et de Pneumologie de Québec, Québec, Canada; 5 INRS-Institut Armand-Frappier, Laval, Québec Canada; 6 Montreal Health Innovation Coordination Center, Montréal, QC Canada; 7 Department of Medicine, Université de Montréal, Montréal, Québec Canada

**Keywords:** Molecular imaging, Adrenomedullin, Pulmonary hypertension, Pulmonary embolism, Nuclear medicine, Peptide

## Abstract

**Purpose:**

The adrenomedullin receptor is densely expressed in the pulmonary vascular endothelium. PulmoBind, an adrenomedullin receptor ligand, was developed for molecular diagnosis of pulmonary vascular disease. We evaluated the safety of PulmoBind SPECT imaging and its capacity to detect pulmonary vascular disease associated with pulmonary hypertension (PH) in a human phase II study.

**Methods:**

Thirty patients with pulmonary arterial hypertension (PAH, *n* = 23) or chronic thromboembolic PH (CTEPH, *n* = 7) in WHO functional class II (*n* = 26) or III (*n* = 4) were compared to 15 healthy controls. Lung SPECT was performed after injection of 15 mCi ^99m^Tc-PulmoBind in supine position. Qualitative and semi-quantitative analyses of lung uptake were performed. Reproducibility of repeated testing was evaluated in controls after 1 month.

**Results:**

PulmoBind injection was well tolerated without any serious adverse event. Imaging was markedly abnormal in PH with ∼50% of subjects showing moderate to severe heterogeneity of moderate to severe extent. The abnormalities were unevenly distributed between the right and left lungs as well as within each lung. Segmental defects compatible with pulmonary embolism were present in 7/7 subjects with CTEPH and in 2/23 subjects with PAH. There were no segmental defects in controls. The PulmoBind activity distribution index, a parameter indicative of heterogeneity, was elevated in PH (65% ± 28%) vs. controls (41% ± 13%, *p* = 0.0003). In the only subject with vasodilator-responsive idiopathic PAH, PulmoBind lung SPECT was completely normal. Repeated testing 1 month later in healthy controls was well tolerated and showed no significant variability of PulmoBind distribution.

**Conclusions:**

In this phase II study, molecular SPECT imaging of the pulmonary vascular endothelium using ^99m^Tc-PulmoBind was safe. PulmoBind showed potential to detect both pulmonary embolism and abnormalities indicative of pulmonary vascular disease in PAH. Phase III studies with this novel tracer and direct comparisons to lung perfusion agents such as labeled macro-aggregates of albumin are needed.

**Clinical trial:**

ClinicalTrials.gov, NCT02216279

**Electronic supplementary material:**

The online version of this article (doi:10.1007/s00259-017-3655-y) contains supplementary material, which is available to authorized users.

## Introduction

Pulmonary hypertension (PH) results from a progressive loss of lung perfusion. Invasive measurement of pulmonary pressure by right heart catheterization is required for precise diagnosis and classification of PH. Further non-invasive evaluation of the biology of lung vasculature and of its alterations in lung vascular disease is currently not available. In the current phase II study, we evaluated the safety and potential of a pulmonary vascular endothelial cell tracer for the detection of pulmonary vascular disease by SPECT imaging.

Adrenomedullin (AM) is a peptide with vasodilatory, anti-inflammatory and anti-proliferative actions [1]. Specific AM receptors are present in the pulmonary vascular endothelium with dense expression in capillaries [2, 3]. The lungs are a major site for circulating AM clearance [4] and pulmonary clearance of AM is reduced in animal models of pulmonary arterial hypertension (PAH) with reduced AM receptor expression [5–7]. Based on these premises, we developed AM derivatives that can be labeled with ^99m^Tc for molecular SPECT imaging of the pulmonary circulation [7]. The lead derivative is called PulmoBind. PulmoBind binds with excellent affinity to the human AM receptor but lacks the hypotensive effects of native adrenomedullin [7]. ^99m^Tc-PulmoBind is effective to detect reduced lung perfusion in the monocrotaline and hypoxia-Sugen models of PAH [6, 7]. In a phase I trial (study PB-01, ClinicalTrials.gov NCT01539889) of healthy human subjects, PulmoBind was safe and resulted in excellent quality SPECT imaging of the lungs [8]. In the current phase II study, we aimed to determine the safety and establish the proof of principle for the use of PulmoBind in the diagnosis of human pulmonary vascular disease.

## Material and methods

The trial was registered at ClinicalTrials.gov (NCT02216279) and conducted in accordance with the amended declaration of Helsinki. All participants signed a written informed consent form. Study subjects were recruited from 3 PH centers: The Montreal Heart Institute, the Jewish General Hospital and the *Institut Universitaire de Cardiologie et de Pneumologie de Québec*.

The primary safety objectives of the study were to determine the safety of PulmoBind in PH and to determine the absence of allergic reaction after a repeated exposure 1 month later in healthy controls. The primary efficacy objective was to determine the capacity of PulmoBind lungs scan for detecting abnormal pulmonary circulation associated with PH.

Control non-smoking participants (*n* = 15) with no evidence of lung disease were recruited after screening tests to rule out PH or any lung disorder (history, physical exam, cardiac ultrasound, chest X-ray and lung function testing) and any significant kidney or liver disease (history, exam, biochemistry). Participants with PH (*n* = 30) were recruited if they had a diagnosis of PAH (idiopathic, heritable, or scleroderma spectrum of disease) or of unoperated chronic thromboembolic PH (CTEPH) in WHO functional class II-III with a 6-min walking distance test of ≥250 m. Diagnosis of PH had to be documented by right heart catheterization performed at any time prior to screening showing a mean pulmonary arterial pressure (mean PAP) >25 mmHg and a pulmonary vascular resistance (PVR) >240 dyn/s · cm with a pulmonary capillary wedge pressure or left ventricular end diastolic pressure ≤15 mmHg. Subjects were excluded if they had significant restrictive lung disease on lung function testing or more than minimal fibrosis on high resolution computed tomography of the chest.

PulmoBind drug substance (American Peptide Company, CA, USA) and diagnostic kits (KABS, St-Hubert, Canada) were produced according to good manufacturing practices with drug substance purity >98%. Each labeling kit contained 18.5 μg (4.33 nmol) of PulmoBind drug substance. After labeling with ^99m^Tc, radiochemical purity was verified by instant thin layer chromatography before each injection. With the subjects lying supine, 15 mCi of ^99m^Tc-PulmoBind was injected in a forearm vein over 5 to 10 seconds. Serial vital signs (heart rate, blood pressure, respiratory rate, temperature and oxygen saturation) were recorded at baseline (about 10 minutes before injection) and at 5 min, 10 min, 15 min, 30 min, and 1 h after PulmoBind injection. Any adverse reaction during the imaging study and up to 30 days after was recorded. The 15 healthy participants underwent a second imaging procedure with ^99m^Tc-PulmoBind 1 month later.

### SPECT-CT image acquisition protocol

Prior to injection, a low dose CT was performed with subjects breathing normally to acquire a thoracic attenuation map. Starting at the time of ^99m^Tc-PulmoBind injection, a 35 min planar dynamic anterior and posterior acquisitions of the lungs were performed with the nuclear camera followed by a 20 min SPECT acquisition. Static planar imaging was again performed after 60 min. The injection syringe was counted before and after injection to determine the exact quantity of drug product administered.

### Image analysis

Image analysis was performed at the Montreal Heart Institute nuclear medicine core lab. Both qualitative and semi-quantitative analyses were performed. For the qualitative analysis, all scans were blindly read by a nuclear medicine specialist (FH) and quoted according to a pre-determined scoring grid to describe the presence of segmental perfusion defects suggestive of pulmonary embolism and for severity as well as extent of heterogeneity of distribution of Pulmobind. The right and left lungs were scored separately. Pulmonary embolism diagnosis was made in the presence of ≥ 2 segmental defects that were pleural-based and triangular-shaped. Perfusion abnormalities not respecting these criteria were considered heterogeneous. Areas of heterogeneity were graded for intensity (mild, moderate, or severe) based on apparent activity, and for their extent of the lung field as mild (<20% of lung), moderate (20% to 60%), and severe (>60%). For the semi-quantitative analysis, tomographic datasets were reconstructed using 3D OSEM (ordered subset expectation maximization) iterative reconstruction algorithm with 3D Gaussian filtering for noise reduction. Semi-automatic 3D regions of interest (ROIs) of the right and left lungs were drawn using ITK-snap 2.2.0 and analyzed using in-house software designed with MATLAB 2013a (Mathworks, Natick, MA, USA). The activity distribution index, a parameter indicative of the distribution of activity intensity that is independent of spatial distribution, was developed to obtain a quantification of the observed heterogeneity. To do so, a frequency histogram of activity intensity per voxel throughout the ROI of each lung was constructed. Voxels were grouped based on their intensity in 15 consecutive clusters from 0% (no activity) to 100% (maximum pixel activity in the ROI), each cluster representing a 6.67% increased activity step. A graph representing the frequency of voxels (equivalent to the percentage of lung volume) was then plotted as a function of the 15 intensity clusters. A reference intensity distribution was constructed from the data obtained in the 15 healthy control subjects. For each participating subject, the intensity distribution was then compared to the normal reference and the percent lung volume with different distribution was determined and defined as the “activity distribution index”. The activity distribution index was measured for the right and left lungs and the maximal value for each subject was analysed.

### Statistical analysis

Clinical parameters at baseline were presented with descriptive statistics, and both groups (PH vs. healthy) were compared using Student t-tests. Safety parameters at Day 1 were presented with descriptive statistics at each time of measurement namely: pre-injection and post-injections (5 min, 10 min, 15 min, 30 min, and 60 min). Maximum variation in each vital sign parameter from the pre-injection value to post-injection values (ex. maximum drop in systolic/diastolic blood pressure, maximum increase in heart rate, etc.) was presented along with the 95% confidence interval for the mean, in healthy and PH participants. Clinical parameters and semi-quantitative parameters of PulmoBind lung uptake at Day 1 were also compared between groups using Student t-tests. Normality of all parameters was tested and log transformation was performed wherever needed. Median, lower, and upper quartiles were presented as appropriate.

Univariate linear regressions were generated in PH subjects with the heterogeneity distribution index as the dependent variable and the following parameters of the severity of PH as the independent variables: type of PH (categorical), 6-min walk distance, WHO functional class (categorical), cardiac ultrasound parameters (pulmonary artery systolic pressure, TAPSE, RVMPI), hemodynamics (mean PAP, PVR, RAP, cardiac output), and NT-proBNP.

Any adverse event was reported and coded by system organ class and body system according to the MedDRA dictionary (version 17.0).

Statistical analyses were conducted at the Montreal Health Innovations Coordinating Center using SAS Version 9.4, after 100% on-site clinical monitoring of the case report forms. All statistical tests were two-sided and performed at a significance level of 0.05.

## Results

The clinical characteristics of healthy controls (*n* = 15) and PH subjects (*n* = 30) are presented in Table 1. There were 23 subjects with PAH (17 idiopathic, three heritable, three scleroderma spectrum of disease) and seven subjects with CTEPH. Most were in WHO functional class II (*n* = 26) with a mean echocardiographic PA systolic pressure of 71 ± 24 mmHg. PH patients were treated with endothelin receptor antagonists (36.7%), phophodiesterase type-5 inhibitors (20.0%), riociguat (26.7%), calcium channel blockers (13.3%), treprostinil (6.7%), and epoprostenol (16.7%). The mean delay between initial PAH diagnosis and inclusion into the trial was 5.0 ± 4.7 years.

**Table 1:** Clinical parameters at baseline

	Healthy controls (*n* = 15)	Pulmonary hypertension (*n* = 30)	*p* value
Age (years)	39 ± 15	54 ± 12	0.0011
Male	10 (66.6%)	9 (30%)	0.0189
Weight (kg)	72 ± 16	70 ± 12	0.6772
Height (cm)	172 ± 10	164 ± 8	0.0063
Body surface area (m^2^)	1.85 ± 0.25	1.79 ± 0.17	0.3154
PH			
Group I, PAH (n)		23	
Group IV, CTEPH (n)		7	
WHO functional class (II/III)		26/4	
6 MWD (m)		473 ± 75	
*FEV_1_ (L)	3.56 (3.36, 4.26)	2.15 (1.85, 2.78)	<0.0001
*FVC (L)	4.40 (4.04, 4.95)	2.99 (2.47, 3.85)	<0.0001
Echocardiography			
PA systolic (mmHg)	25 ± 4	71 ± 24	<0.0001
TAPSE (mm)	26.0 ± 3.6	20.4 ± 3.8	<0.0001
RVMPI	0.21 ± 0.09	0.57 ± 0.30	<0.0001
Hemodynamic			
Mean PA pressure (mmHg)		46 ± 12	
PVR (Wood units)		7.2 ± 4.0	
Right atrial pressure (mmHg)		7.8 ± 4.4	
Cardiac output (L/min)		5.1 ± 1.2	
*eGFR (ml/min/1.73 m^2^)	96 (90, 111)	80 (73, 89)	0.0008
*NT-proBNP (ng/L)	29 (11, 56)	141 (78, 532)	<0.0001

Values are mean ± sd , median (Q1, Q3) or n (%)

PH pulmonary hypertension; PAH pulmonary arterial hypertension; CTEPH chronic thromboembolic pulmonary hypertension; WHO World Health Organization; 6 MWD 6-min walking distance; FEV _1_ forced expiratory volume in 1 s; FVC forced vital capacity; PA pulmonary artery; TAPSE tricuspid annulus plane systolic excursion; RVMPI right ventricular myocardial performance index; PVR pulmonary vascular resistance; eGFR estimated glomerulare filtration rate; NT-proBNP N-terminal pro-brain natriuretic peptide.

* *p*-value based on log-transformed data

All subjects completed the study and there were no serious adverse events. The overall mean labeling efficiency of ^99m^Tc-PulmoBind at Day 1 was 98% ± 2%. The mean activity of ^99m^Tc-PulmoBind injected was 14.6 ± 1.82 mCi. This represents a mean amount of PulmoBind drug substance per injection of 6.78 μg (1.59 nmole). There was one mild adverse event possibly related to study drug, as 1 PH participant reported a transient sensation of metallic taste following PulmoBind injection. In two participants (one healthy and one PH), there was infiltration of PulmoBind in forearm tissues during injection. These subjects were observed and no local reaction occurred. They were both re-injected (one on same day and one the day after) without any adverse event. Serial vital signs at baseline and at each time point following injection of PulmoBind are presented in Table 2 together with the mean maximum variations, irrespective of the time point. There was a mild decrease of systolic and diastolic blood pressures over the first 15 min that remained stable for the duration of the study (1 h). There was no clinically significant change in heart rate, respiratory rate, temperature, and oxygen saturation.

**Table 2:** Safety parameters at Day 1

	Systolic BP (mmHg)		Diastolic BP (mmHg)		Heart Rate (beats/min)		Respiratory Rate (breaths/min)		O_2_ Saturation (%)		Temperature (°C)	
	Controls	PH	Controls	PH	Controls	PH	Controls	PH	Controls	PH	Controls	PH
Baseline												
∼ −10 min	116 ± 11	116 ± 15	70 ± 9	72 ± 8	59 ± 7	69 ± 13	15 ± 3	17 ± 2	99 ± 0.9	95 ± 2.7	36.6 ± 0.2	36.5 ± 0.3
5 min	113 ± 11	113 ± 14	69 ± 6	71 ± 8	58 ± 7	68 ± 11	14 ± 3	18 ± 2	99 ± 1.1	94 ± 3.1	36.6 ± 0.1	36.7 ± 0.1
10 min	110 ± 10	110 ± 14	67 ± 6	68 ± 9	59 ± 8	68 ± 12	14 ± 2	18 ± 2	98 ± 1.4	93 ± 3.5	36.6 ± 0.2	36.7 ± 0.1
15 min	109 ± 9	109 ± 14	65 ± 7	67 ± 9	59 ± 7	69 ± 12	13 ± 2	18 ± 2	99 ± 1.3	93 ± 3.6	36.6 ± 0.1	36.7 ± 0.2
30 min	111 ± 11	109 ± 13	66 ± 7	66 ± 10	61 ± 7	70 ± 12	14 ± 3	18 ± 2	99 ± 1.4	93 ± 3.8	36.6 ± 0.1	36.6 ± 0.3
60 min	113 ± 9	114 ± 14	68 ± 8	69 ± 8	61 ± 9	70 ± 12	15 ± 2	18 ± 2	99 ± 0.7	95 ± 3.5	36.6 ± 0.1	36.6 ± 0.3
Maximum change	−8.7 (−12.0, −5.3)	−11.2 (−14.3, −8.0)	−6.7 (−8.8 -4.5)	−8.6 (−10.8, −6.3)	4.9 (2.5, 7.4)	3.7 (1.7, 5.8)	0.13 (−0.78, 1.04)	0.20 (−0.40, 0.80)	−1.3 (−2.0, −0.5)	−2.9 (−3.6, −2.1)	0.11 (0.03, 0.20)	0.08 ( −0.01, 0.18)

Values are mean ± sd or mean (95% CI)

**Fig. 1:**
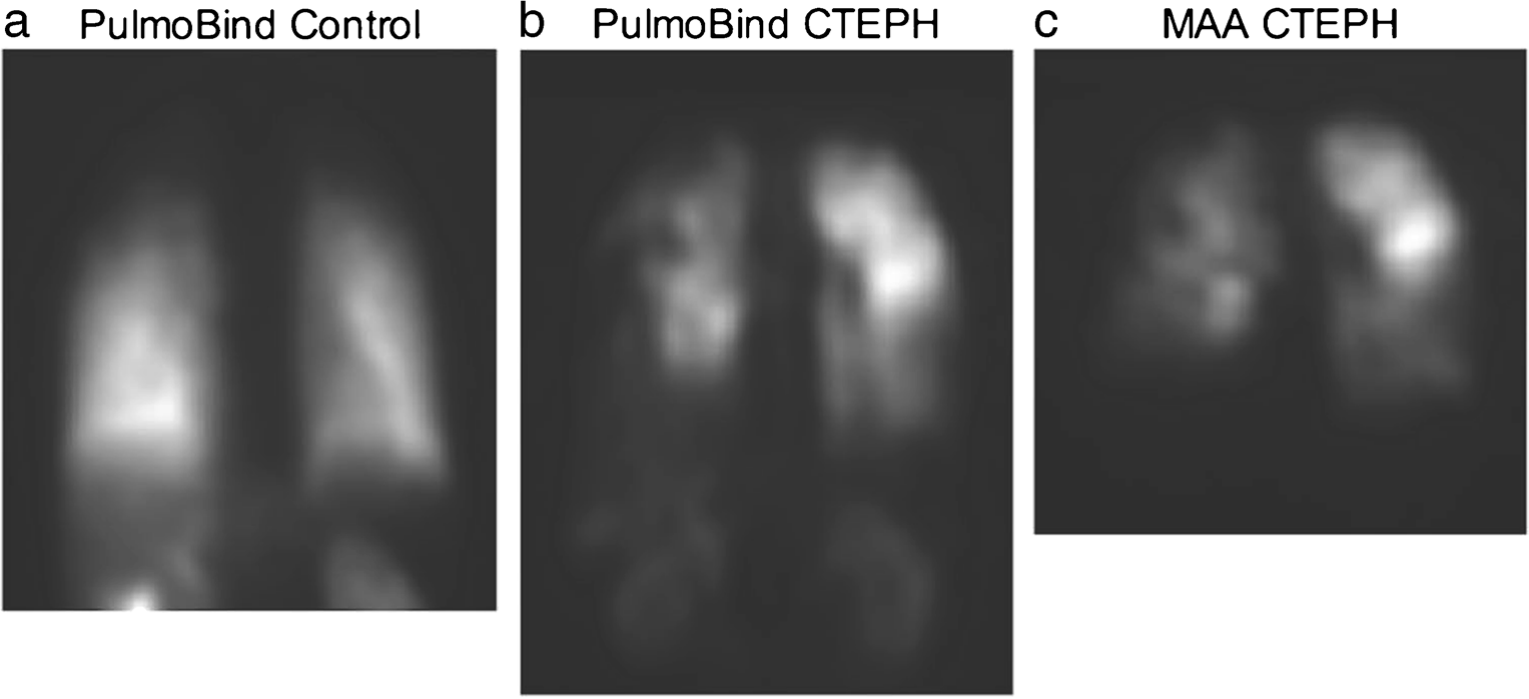
^99m^Tc-PulmoBind lung scan in CTEPH. ^99m^Tc-PulmoBind lung scans in a healthy control (**a**) and in a subject with CTEPH (**b**) compared to the MAA lung scan of the same CTEPH subject 5 years earlier (c)

Peak PulmoBind uptake by the lungs was 63% ± 5% of the injected dose in healthy controls and was similar in PH subjects although with greater variability at 60% ± 12% (Table 3). Uptake at 30 min and lung half-life of PulmoBind were also not statistically different. The time to peak lung uptake was however longer in the PH group (5.1 ± 1.1 min) compared to the healthy controls (4.3 ± 0.8 min, *p* = 0.0194). Blinded qualitative analysis of PulmoBind lung scans resulted in the identification of segmental perfusion defects compatible with pulmonary embolism in nine PH subjects, but in none of the healthy controls. For CTEPH subjects, 7/7 were interpreted as having segmental perfusion defects compatible with pulmonary embolism, and for PAH it was 2/23 subjects. A subject with CTEPH is shown in Fig. 1 compared to the MAA lung scan obtained previously (5 years before) in the same subject and to a healthy control. In that subject and despite the long delay between exams, MAA and PulmoBind detect multiple segmental perfusion defects of similar sizes and distribution.

**Table 3:** Lung PulmoBind uptake parameters at day1

	Healthy controls (*n* = 15)	Pulmonary hypertension (*n* = 30)
Peak uptake (%)	63 ± 5	60 ± 12
Time to peak uptake (min)	4.3 ± 0.8	5.1 ± 1.1*
Uptake at 30 min (%)	49 ± 5	50 ± 11
Lung half-life (min)	71.1 ± 7.8	70.0 ± 11.8

*p <0.05 PH vs. Healthy controls

**Fig. 2:**
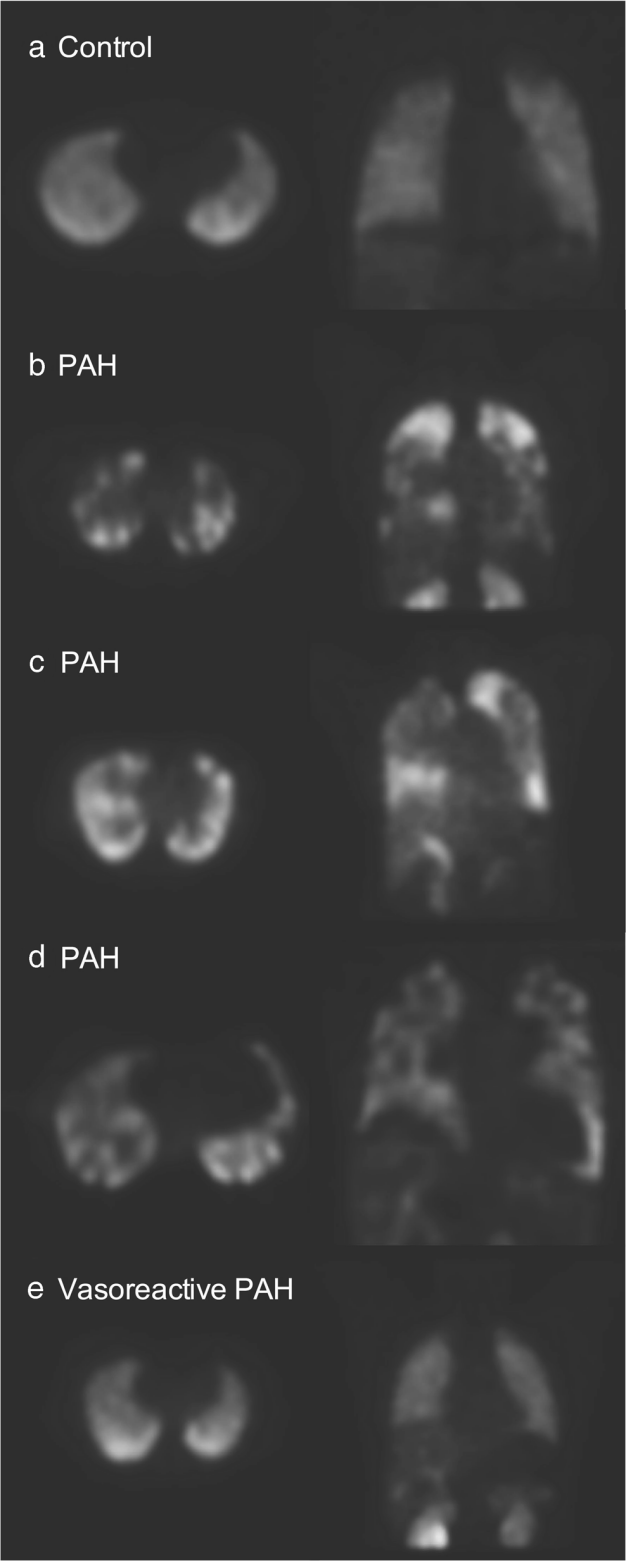
^99m^Tc-PulmoBind lung scan in PAH. ^99m^Tc-PulmoBind lung SPECT in a control subject (**a**), in three subjects with PAH (**b**,**c**,**d**) and in one subject with idiopathic reversible PAH (**e**)

**Fig. 3:**
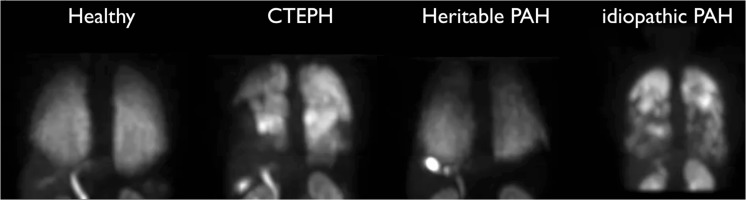
^99m^Tc-PulmoBind lung scan in various types of PH. ^99m^Tc-PulmoBind lung SPECT in a control subject and in subjects with CTEPH, heritable PAH and idiopathic PAH. Also available online as a supplemental video


There was mild heterogeneity of distribution of PulmoBind, mostly of mild extent, in about 30% of healthy control lungs (Table 4). None of the healthy controls demonstrated more than mild heterogeneity. Moderate to severe heterogeneity of moderate to severe extent was present in about 50% of subjects with PH and was unevenly distributed between the right and left lungs (Table 4). Examples of abnormal PulmoBind lung scans in subjects with PAH are shown in Fig. 2. Examples of exams in various types of PH are shown in Fig. 3 and in a video (online supplemental material). Compared to the healthy controls, areas of prominently reduced activity are evident in both lungs. The deficits are heterogeneously distributed within one lung and between the right and left lungs with some areas that are apparently spared showing normal or even increased activity. No specific patterns of distribution were found with variations between subjects. Interestingly, in the only subject with vasodilator-responsive PAH that was included into the study, a completely normal distribution of PulmoBind was found (bottom scan of Fig. 2).

**Table 4:** Qualitative evaluation of lung PulmoBind uptake at Day 1

	Healthy controls (*n* = 15) right left		PH (*n* = 30) right left	
Heterogeneity present (*n* (%))	5 (33.3%)	3 (20.0%)	14 (46.7%)	15 (50.0%)
Severity (*n*)				
Mild	5	3	6	6
Moderate	0	0	7	8
Severe	0	0	1	1
Extent (*n*)				
Mild	2	1	0	1
Moderate	3	2	10	10
Severe	0	0	4	4

(M4V 6817 kb)


**Fig. 4:**
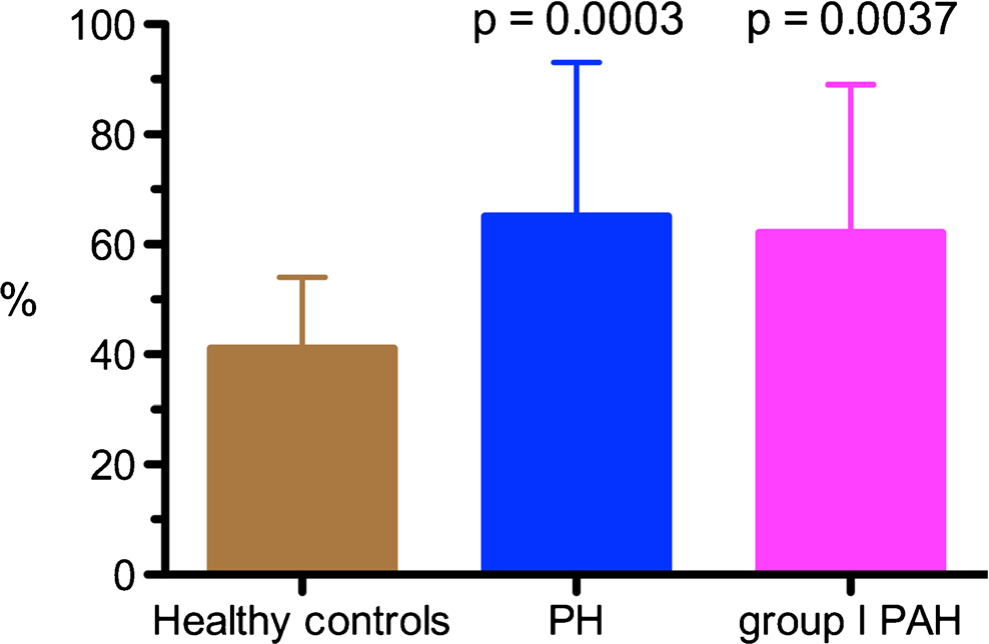
Activity distribution index. The activity distribution index, a parameter indicative of heterogeneity of lung ^99m^Tc-PulmoBind distribution is shown in healthy controls (*n* = 15), in all PH subjects of the trial (*n* = 30) and in the PAH subgroup only (*n* = 23). Values are mean with 95% confidence intervals

**Fig. 5:**
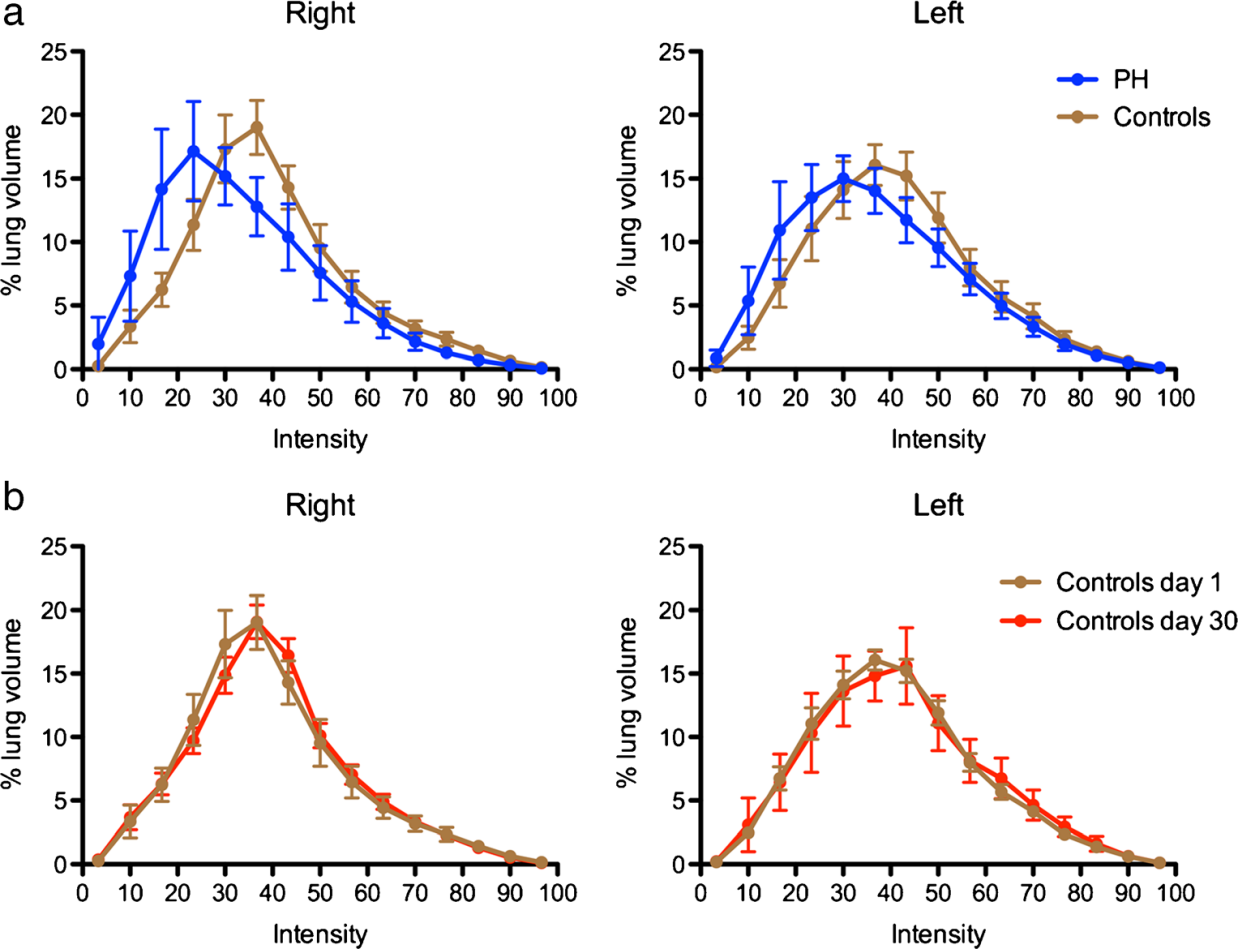
Frequency distribution of lung ^99m^Tc-PulmoBind activity. The percentage of lung volume per voxel intensity is plotted for PH subjects and controls (**a**) and for controls on day 1 and day 30 (**b**) for the right and left lungs. Values are mean with 95% confidence intervals

We developed the activity distribution index; a semi-quantitative parameter indicative of the heterogeneity of PulmoBind lung activity for each individual compared to a reference distribution derived from our healthy control population. The activity distribution index in healthy controls was 41% ± 13% (Fig. 4). The value was higher in all PH subjects at 65% ± 28% (*p* = 0.0003), as well as in the PAH only subjects at 62% ± 27% (*p* = 0.0037). The frequency histogram of lung voxels intensity from which the activity distribution index is computed is shown in Fig. 5 for healthy controls at day 1 and day 30 as well as for PH subjects. In controls, the activity is distributed following a standard bell curve with excellent reproducibility of results after a repeated exam. In PH subjects, the activity distribution is skewed to lower intensity voxels. The activity distribution index was not correlated with parameters of PH severity.

## Discussion

There is currently no imaging test that can provide direct information on the biologic properties of the pulmonary vascular endothelium [9]. It is generally recognized that endothelial dysfunction is an initiating and/or perpetuating event in pulmonary vascular diseases [10]. Pulmonary vascular diseases can lead to PH, a devastating condition often diagnosed late in the evolution process, as a substantial proportion of the pulmonary vascular bed must be affected before an elevation of pulmonary pressures is clinically detectable at rest. We developed PulmoBind, an AM receptor ligand, for the diagnosis of pulmonary vascular disease [5, 7, 8, 11, 12]. PulmoBind specifically binds to the human AM receptor that is densely expressed in the pulmonary vascular endothelium, mostly in capillaries [2, 3]. The pulmonary circulation is the primary site for circulating AM clearance [4, 12]. The level of expression and activity of the AM receptor will therefore modify PulmoBind uptake by the lungs. In this phase II human study, we evaluated the safety of PulmoBind administration in subjects with PH and its capacity to detect changes in the pulmonary circulation associated with PH.

In a previous phase I trial in 20 healthy subjects, we demonstrated that ^99m^Tc-PulmoBind was safe and well tolerated and resulted in lung imaging of excellent quality [8]. Similarly, ^99m^Tc-PulmoBind injections did not cause any serious adverse events in the 15 healthy controls and 30 PH subjects included in the current study. In two subjects, there was accidental local infiltration in forearm tissues with no local reaction. There were no clinically significant variations of vital signs. Compared to baseline, a mild decrease in systemic blood pressure was observed in the first 15 min after injection that was maintained for the duration of the study (1 h). This decrease is compatible with the normal physiological variation expected during the experimental procedure: the subjects were comfortably lying supine in a quiet environment and baseline measurements were obtained about 10 min prior to PulmoBind injection. There is indeed a normal decrease in systemic blood pressure measurement that stabilizes after 15 min of rest in seated or supine subjects [13, 14]. The mean lung scan dose of PulmoBind in humans is of 6.78 μg, an amount with a safety margin of at least 100X in animal studies compared to native AM [7]. Furthermore, repeated injection of ^99m^Tc-PulmoBind in the healthy controls did not cause any adverse reaction.

The efficacy analysis included a blinded qualitative evaluation as well as semi-quantitative analysis of ^99m^Tc-PulmoBind lung SPECT. Segmental perfusion defects compatible with CTEPH were found in 7/7 subjects with CTEPH and in 2/23 subjects with PAH. Although limited to perfusion imaging without simultaneous lung ventilation studies, this suggests that ^99m^Tc-PulmoBind SPECT imaging could be further tested for the diagnosis of CTEPH in subjects with PH. This finding also provides impetus to evaluate PulmoBind for the diagnosis of acute pulmonary thrombo-embolic disease with direct comparison to labeled MAA and angio-CT.

About 50% of PH subjects had moderate to severe lung heterogeneity of moderate to severe extent while controls had no more than mild heterogeneity. No specific patterns of heterogeneity could be discerned as the distribution varied within each lung and between the right and left lungs. The abnormal distribution patterns also varied between individuals (Fig. 2). These data, obtained in WHO functional class II PH, are consistent with histologic studies from advanced PH transplant candidates demonstrating that the pathologic processes in PH are not homogeneously distributed [15]. We found that some regions demonstrated absent or reduced uptake of ^99m^Tc-PulmoBind while others had normal or even seemed to have increased uptake explaining that the overall mean lung uptake was similar to healthy controls, although with much wider variability. While it is certain that non-perfused regions will have no uptake, as confirmed in the CTEPH subjects, we cannot exclude that some perfused regions with endothelial dysfunction may have no or reduced AM receptor expression leading to reduced activity. Moreover, because ^99m^Tc-PulmoBind is a very small molecular tracer, it probably can detect small areas of non-perfused lung associated to the microthrombi observed in PAH [15]. Conversely, increased expression of AM receptors in non-affected lung regions could account for apparent increase in uptake. Although speculative, some lung regions may indeed compensate by recruiting vasculature and increasing the expression of the AM receptor. Temporal evaluation of these processes in various types of PH could provide novel pathophysiologic insights. Also remarkable is the normal perfusion pattern in our vasodilator-responsive idiopathic PAH patient. This type of patient represents a rare instance of idiopathic PAH where hemodynamics can normalize with therapy, and the normal perfusion we found is consistent with this phenotype.

To semi-quantitatively evaluate the distribution of ^99m^Tc-PulmoBind, we developed the activity distribution index. An increase in the index is indicative of heterogeneity of distribution compared to the mean of healthy controls. The activity distribution index was markedly increased in all PH subjects as well as in the PAH subgroup only. Although this study is limited in size with mostly WHO class II subjects, we explored potential correlation of the activity distribution index with parameters of severity of PH and found no correlation. Larger sample size studies will be required to determine if this parameter could be a useful biomarker of disease severity and potentially of response to therapy. We found that the time to peak lung uptake of PulmoBind was significantly increased in PH subjects. This could be related to longer pulmonary transit times associated with lower cardiac outputs in these subjects. We cannot, however, exclude that this difference could be caused by other non-pathologic factors since the controls and PH subjects had different demographics with more women and older subjects in the PH group. This “proof of concept” and safety phase II study has several inherent limitations, in particular its small sample size, the variable time since PH diagnosis and the lack of a comparison to a perfusion agent. Larger studies including more subtypes of PH will be necessary to determine if specific patterns of distribution could be indicative of specific etiologies. Direct comparative studies to labeled MAA and angio-CT are required to determine if PulmoBind provides any additional advantage to these agents.

The major strength of this study are that it demonstrates for the first time that we can safely image human pulmonary vascular disease by using a vascular endothelial cell tracer. Other molecular tracers were previously used to image the pulmonary circulation and were recently reviewed in detail [9]. Many of these tracers also targeted properties of the pulmonary vascular endothelium, but none have been further developed for clinical use despite some encouraging results.

## Conclusion

In this phase II study, molecular SPECT imaging of the pulmonary vascular endothelium using ^99m^Tc-PulmoBind was safe. PulmoBind showed potential to detect both pulmonary embolism and abnormalities indicative of pulmonary vascular disease in PAH. However, it is unclear whether PulmoBind is a perfusion tracer only or if it adds functional information on adrenomedullin receptors in PH. The potential clinical utility of this agent should now be tested in phase III trials with direct comparison to labeled MAA and angio-CT.

AM, adrenomedullin; CT, computed tomography; CTEPH, chronic thromboembolic pulmonary hypertension; MAA, macroaggregates of albumin; PA, pulmonary artery; PAH, pulmonary arterial hypertension; PH, pulmonary hypertension; ROI, region of interest; SPECT, single photon emission computed tomography; Tc, technetium
